# Conditional expression of flagellar motility, curli fimbriae, and biofilms in Shiga toxin- producing *Escherichia albertii*

**DOI:** 10.3389/fmicb.2024.1456637

**Published:** 2024-09-09

**Authors:** Michelle Qiu Carter, Diana Carychao, Rebecca L. Lindsey

**Affiliations:** ^1^Produce Safety and Microbiology Research Unit, U.S. Department of Agriculture, Agricultural Research Service, Western Regional Research Center, Albany, CA, United States; ^2^Enteric Diseases Laboratory Branch, Centers for Disease Control and Prevention, Atlanta, GA, United States

**Keywords:** *Escherichia albertii*, foodborne pathogen, biofilm, adhesins, fimbriae, flagella, motility

## Abstract

*Escherichia albertii* is an emerging foodborne pathogen. We previously reported that some avian Shiga toxin-producing *E. albertii* strains exhibited higher or comparable cytotoxicity in Vero-d2EGFP cells with several enterohemorrhagic *E. coli* (EHEC) outbreak strains. To better understand the environmental persistence of this pathogen, comparative genomics and phenotypic assays were applied to assess adhesion capability, motility, and biofilm formation in *E. albertii*. Among the 108 adherence-related genes, those involved in biogenesis of curli fimbriae, hemorrhagic *E. coli* pilus, type 1 fimbriae, and Sfm fimbriae were conserved in *E. albertii*. All 20 *E. albertii* strains carried a complete set of primary flagellar genes that were organized into four gene clusters, while five strains possessed genes related to the secondary flagella, also known as lateral flagella. Compared to EHEC strain EDL933, the eight chemotaxis genes located within the primary flagellar gene clusters were deleted in *E. albertii*. Additional deletion of motility genes *flhABCD* and *motBC* was identified in several *E. albertii* strains. Swimming motility was detected in three strains when grown in LB medium, however, when grown in 5% TSB or in the pond water-supplemented with 10% pigeon droppings, an additional four strains became motile. Although all *E. albertii* strains carried curli genes, curli fimbriae were detected only in four, eight, and nine strains following 24, 48, and 120 h incubation, respectively. Type 1 fimbriae were undetectable in any of the strains grown at 37°C or 28°C. Strong biofilms were detected in strains that produced curli fimbriae and in a chicken isolate that was curli fimbriae negative but carried genes encoding adhesive fimbriae K88, a signature of enterotoxigenic *E. coli* strains causing neonatal diarrhea in piglets. In all phenotypic traits examined, no correlation was revealed between the strains isolated from different sources, or between the strains with and without Shiga toxin genes. The phenotypic variations could not be explained solely by the genetic diversity or the difference in adherence genes repertoire, implying complex regulation in expression of various adhesins. Strains that exhibited a high level of cytotoxicity and were also proficient in biofilm production, may have potential to emerge into high-risk pathogens.

## 1 Introduction

*Escherichia albertii*, an emerging foodborne pathogen, is the most divergent lineage among the other *Escherichia* species and clades (Walk et al., 2009; Ooka et al., 2015). Due to similar biochemical properties and possession of the intimin gene located on the locus of enterocyte effacement (LEE) pathogenicity island, many *E. albertii* isolates have been misidentified as enteropathogenic *E. coli* (EPEC), or enterohemorrhagic *E. coli* (EHEC) (Ooka et al., 2012). *E. albertii* causes diarrhea, abdominal pain, and high fever in humans, although bacteremia and extraintestinal infections were also reported (Gomes et al., 2020). The well-known virulence factors in *E. albertii* include LEE encoded intimin and its Tir receptor, responsible for the initial adherence of pathogen cells to the host epithelial cell surfaces, as well as the LEE-encoded type three secretion system (T3SS) and the effector proteins. Other common virulence factors include Shiga toxin (Stx), cytolethal distending toxin (CDT), type six secretion systems (T6SS), and the vacuolating autotransporter toxin Vat (Carter et al., 2023a). Sporadic infections and outbreaks of foodborne gastroenteritis caused by *E. albertii* have been reported worldwide (Konno et al., 2012; Ooka et al., 2013; Ori et al., 2018; Masuda et al., 2020; Bengtsson et al., 2023; Iguchi et al., 2023). Transmission of *E. albertii* is thought to occur via contaminated food or water although in most outbreaks the transmission vehicles were not identified (Masuda et al., 2020; Muchaamba et al., 2022).

Growing evidence supports that *E. albertii* has a wide habitat range. *E. albertii* strains have been isolated from domestic and wild animals, various foods, and aquatic environments (Muchaamba et al., 2022). Among the reported animal hosts, birds appear to be one of the main reservoirs/carriers (Oaks et al., 2010; Hinenoya et al., 2021; Hinenoya et al., 2022; Wang et al., 2022; Barmettler et al., 2023; Xu et al., 2024). Presence of *E. albertii* in various water bodies and food products including chicken, pork, duck meat, mutton, and oysters has been reported (Felfoldi et al., 2010; Maheux et al., 2014; Lindsey et al., 2015; Maeda et al., 2015; Wang et al., 2016; Arai et al., 2022), however, little is known about the contamination routes and the environmental prevalence and persistence of this emerging foodborne pathogen. A recent study investigating the survival of *E. albertii* in foods and water revealed that *E. albertii* grew faster in chicken than in pork or in oysters but had low viability in warm environmental water (Hirose et al., 2024). Induction of flagellar biosynthesis and swimming motility was observed in some strains when cells were exposed to hypoosmotic pressure or at ambient temperature, suggesting a role of flagellar motility in the survival of *E. albertii* in aquatic environments (Ikeda et al., 2020).

Biofilm is a common microbial lifestyle in natural environments (Watnick and Kolter, 2000). Compared with planktonic cells, biofilm-associated cells are better at coping with environmental stresses and have increased resistance to toxic substances including antibiotics and chemical sanitizers. Therefore, biofilm formation by enteropathogenic bacteria would increase their survival and persistence in natural environments and may serve as a source of contamination. Biofilm formation involves multiple steps, including initial surface contact, transient association, attachment, maturation, and dispersion (O’Toole et al., 2000). Numerous bacterial adherence factors including surface adhesive appendages and autotransporter proteins play a role in biofilm formation. In *E. coli* K-12 strains, flagellar motility, curli fimbriae, as well as FimH adhesin were found to be important for initial surface contact and attachment. Additionally, flagellar motility was found playing a role in biofilm dispersion (Pratt and Kolter, 1998; Reisner et al., 2003; Karatan and Watnick, 2009).

Shiga toxin-producing *E. coli* (STEC) produces diverse fimbrial and nonfimbrial adhesins that facilitate the attachment to and/or colonization by STEC cells in diverse ecological niches (McWilliams and Torres, 2014; Vogeleer et al., 2014). In STEC O157:H7 strains, curli fimbriae were found to mediate binding to, and invasion of epithelial cells, and promote the attachment of pathogens to plant and abiotic surfaces (Gophna et al., 2001; Fink et al., 2012; Carter et al., 2016). The hemorrhagic *E. coli* pilus (HCP), originally identified in STEC O157:H7 as a colonization factor (Xicohtencatl-Cortes et al., 2007), contributed to the biofilm formation of STEC O157:H7 strains on abiotic surfaces (Xicohtencatl-Cortes et al., 2009). In *E. coli* and other enteric pathogens, expression of type 1 fimbriae is controlled by a phase variation mechanism, which reversibly switches between the “ON” and “OFF” state of *fim* genes transcription (Abraham et al., 1985). This switch is mediated by an invertible DNA element, *fimS*, and two site-specific recombinases. Inversion of *fimS* abolishes the transcription of *fimA*, which encodes the major subunit of type 1 fimbriae. Expression of type 1 fimbriae were detected in STEC non-O157 strains, but not in O157:H7 strains (Roe et al., 2001). In STEC O157:H7 strains, transcription of *fimA* is locked at the “OFF” state due to a 16-bp deletion within the *fimS* (Iida et al., 2001). Type 1 fimbriae contributed to the attachment of STEC cells to abiotic surfaces in a O128:H2 strain and contributed to the biofilm formation when the *fim* genes of the STEC O157:H7 strain Sakai were expressed in a nonpathogenic *E. coli* strain (Cookson et al., 2002; Elpers and Hensel, 2020).

Knowledge about environmental persistence of *E. alberti* is scarce. Biofilm formation by *E. albertii* was reported in only a few clinical strains at 37°C although the efficiency of biofilm formation was much lower than that of *E. coli* strain 042 (Lima et al., 2019). Understanding prevalence and persistence of *E. albertii* in nonhost environments will provide valuable information for risk assessment and to bridge gaps in understanding the epidemiology of this emerging human pathogen. We previously reported genomic features and virulence genes repertoire of Shiga toxin-producing *E. albertii* strains isolated from wild birds in an agricultural region in California and revealed that some bird strains exhibited higher or comparable cytotoxicity with several EHEC outbreak strains (Carter et al., 2023a). To gain insight into the persistence of *E. albertii* in nonhost environments, we systematically evaluated the adhesion capability and several phenotypic traits known to contribute to bacterial biofilm formation in a set of *E. albertii* avian and clinical strains. Our study revealed great genetic diversity in genes encoding fimbrial and nonfimbrial adhesins in *E. albertii* as well as vast strain variations in expression of curli fimbriae, swimming motility, and in biofilm formation. Our study provides a foundation into further understanding how *E. albertii* senses and responds to environmental stimuli for improved survival in the changing environments.

## 2 Materials and methods

### 2.1 Bacterial strains and growth media

Bacterial strains and their sources are listed in Table 1. The complete genome sequences of *E. albertii* strains were reported previously (Carter et al., 2023a). The strains were grown routinely in Luria-Bertani (LB) broth (10 g tryptone, 5 g yeast extract, and 5 g NaCl per liter) unless noted.

**TABLE 1:** *E. albertii* strains used in this study and the motility test.

Strains	Sources/Year of isolation	GenBank BioSample Number	^a^Swimming Motility		
*stx*_2*f*_-positive *E. albertii*			LB	5% TSB	Pond water with 10% pigeon droppings
RM9973	American crow (*Corvus brachyrhynchos*)/2009	SAMN12620691	−	+	+
RM9974	American crow (*Corvus brachyrhynchos*)/2009	SAMN12620692	−	−	−
RM9976	American crow (*Corvus brachyrhynchos*)/2009	SAMN12620693	−	+	+
RM10507	Brown-headed cowbird (*Molothrus ater*)/2009	SAMN12620694	+	+	+
RM10705	Brown-headed cowbird (*Molothrus ater*)/2009	SAMN12620697	+	+	+
RM15112	Oregon Junco (*Junco hyemalis*)/2011	SAMN12620700	−	−	−
RM15113	Oregon Junco (*Junco hyemalis*)/2011	SAMN12620701	−	−	−
RM15114	Oregon Junco (*Junco hyemalis*)/2011	SAMN12620702	−	−	−
RM15115	White-Breasted Nuthatch (*Sitta carolinensis*)/2011	SAMN12620703	−	−	−
RM15116	Oregon Junco (*Junco hyemalis*)/2011	SAMN12620704	−	−	−
2011C-4180	Human/2011	SAMN03019926	−	−	−
2012EL-1823B	Human/2012	SAMN04498560	−	−	−
2014C-4015	Human/2014	SAMN04505646	−	−	−
2014EL-1348	Human/2014	SAMN04505647	−	−	−
*stx*-negative *E. albertii*					
2014C-4356	Chicken Carcass/2009	SAMN07159041	−	+	+
05-3106	Human/2005	SAMN08199278	−	+	+
07-3866	Human/2007	SAMN07159045	+	+	+
54-2045 (NCTC 9362)	Human/1954	SAMN09534374	−	−	−
2010C-3449	Human/2010	SAMN07159044	−	−	−
2013C-4143	Human/2013	SAMN08172567	−	−	−

^a^Swimming motility was observed after incubation at 30°C for three days.

### 2.2 Sequence analysis

The flagellar genes in *E. albertii* strains were identified by using BLASTn searches with the flagellar genes of the *E. coli* K-12 sub-strain MG1655 and the EHEC strain EDL933 (Supplementary Table 1). Additional flagellar genes were identified from the *E. albertii* genome annotations as described previously (Carter et al., 2023a) (Supplementary Table 2). *E. coli* genes related to fimbriae and pili biogenesis and genes encoding protein adhesins (Supplementary Table 3) were used as queries of BLASTn to identify homologs of adherence-related genomic loci in *E. albertii* strains. The BLASTn was performed in Geneious Prime^®^ with a threshold of 65% for gene coverage and 70% or 25% for sequence identity at nucleotides or amino acids level, respectively. Homologs of each gene or the entire operons were extracted from the corresponding bacterial genomes. DNA sequences were aligned using Clustal Omega in Geneious Prime^®^ (2024.0.3) and neighbor-joining consensus trees were constructed with the following parameters: Genetic Distance Model, Jukes-Cantor; Resampling Method, bootstrap; and number of replicates, 10,000.

### 2.3 Motility tests

Swimming motility was examined for each strain grown on soft agar (0.25%) in rich medium (LB), diluted TSB (5%), and sterile pond water containing 10% pigeon droppings as described previously (Murakami et al., 2020) with modification. The pond water was collected from a public accessible creek in Albany, California (37°53′43.86′′N, 122°18′16.68′′W). The pigeon droppings were collected near a train station in El Cerrito, California (37°54′9.63′′N, 122°17′56.17′′W). To prepare the 10% pigeon-droppings suspension, pigeon droppings were first suspended in nine volumes of pond water and then filtered through a 0.22-μm filter followed by adding agar to 0.25% prior to autoclaving. Single colonies of each *E. albertii* strain were point-inoculated on soft agar plates using sterile toothpicks. The plates were incubated at 30°C for three days prior to observing the motility.

### 2.4 Detection of curli fimbriae

Curli fimbriae were examined by growing each strain at 26°C for 1, 2, and 5 days on Congo Red indicator (CRI) plates, consisting of LB agar plates without sodium chloride (LBNS) and supplemented with 40 μg/ml of Congo Red dye and 10 μg/ml of Coomassie Brilliant Blue, as described previously (Carter et al., 2011). Curli-producing strains were indicated by red colonies whereas curli-deficient strains were indicated by white colonies on CRI plates.

### 2.5 Detection of type 1 fimbriae

Production of type 1 fimbriae was examined by hemagglutination for each strain grown in LBHS broth statically at 37°C or in LBNS broth statically at 28°C for two days. Cells were collected by centrifugation at 8,000 g for 3 min and resuspended in 1x PBS buffer at a final concentration about 3 x 10^8^ cells/ml. Fifty μl of bacterial suspension was then mixed with 50 μl of guinea pig red blood cells (Innovative Research Inc) at room temperature in the presence or absence of 1% D mannose as previously described (Biscola et al., 2011). *E. coli* strain DH5a was used as a positive control and EHEC strain EDL933 was used as a negative control.

### 2.6 Biofilm formation and quantification

Biofilm assays were carried out as described previously (Carter et al., 2023b). Briefly, overnight cultures of *E. albertii* grown in LB at 37°C were inoculated in LBNS broth at a final concentration of 1x10^6^ cells/ml. One ml of inoculated LBNS broth was aliquoted into a borosilicate glass tube and then incubated statically at 28°C for 1, 2, and 5 days. At the end of each incubation, the planktonic cells were removed carefully, and the tubes were rinsed twice with one ml sterile distilled water and then stained with one ml 0.1% crystal violet at room temperature for 30 min. The dye was then removed gently, and the tubes were washed twice with sterile distilled water. The crystal violet that bound to the glass tube was solubilized in 0.5 ml of 33% acetic acid and the absorbance was determined at 570 nm using a microplate reader (SpectraMax 340; Molecular Devices, Sunnyvale, CA). Tubes with uninoculated media served as negative controls. Each data set was the average of results from at least three biological replicates. All data were first evaluated for normal distribution by the Shapiro-Wilk test using Graph Pad Prism 10 Version 10.2.3 (Dotmatics). The differences in biofilm formation, represented by the absorbance at 570 nm, among the strains were assessed by the adjusted *P*-value of the Tukey’s multiple comparisons test after a One-way ANOVA test (*P* ≤ 0.05). Similarly, the differences in biofilm formation of each strain at various incubation times were assessed by the adjusted *P*-value of the Tukey’s multiple comparisons test after a One-way ANOVA test.

## 3 Results

### 3.1 *E. albertii* flagellar genes

**FIGURE 1:**
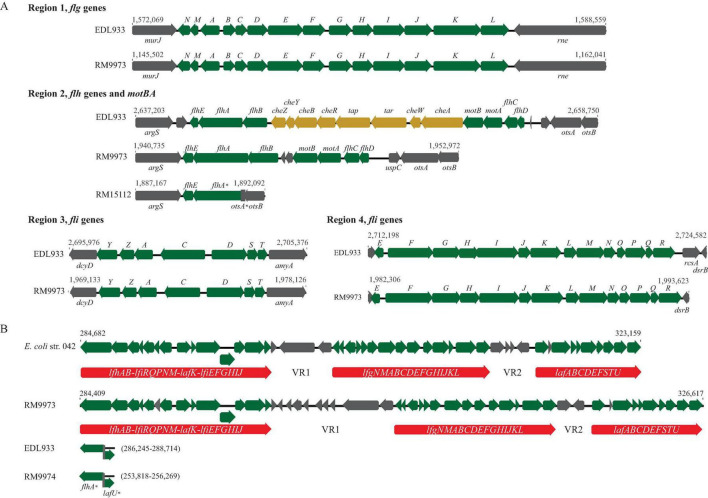
Sequence analyses of *E. albertii* flagellar genes. **(A)** Gene organization and genomic locations of the primary flagellar genes. Numbers indicate the corresponding chromosomal positions of the four flagellar gene clusters in *E. coli* O157:H7 strain EDL933 and *E. albertii* avian strains RM9973 and RM15112. Green arrows represent the flagellar genes detected in *E. albertii* and grey arrows represent the bordering genes or the hypothetical genes. Yellow arrows represent *E. coli* genes that are missing in *E. albertii* strains. **(B)** Gene organization and genomic locations of the secondary flagellar genes (Flag-2). Numbers indicate the corresponding chromosomal positions in EAEC strain 042 and avian strain RM9973. The three flagellar gene clusters are indicated by the red arrows. In the strains lacking a Flag-2, such as EDL933 and RM9974, the corresponding chromosomal sites were uniformly occupied by the two truncated genes, *flhA* and *lafU*. Genes labeled with an “*” indicate those carrying mutations within the coding sequences.

Of the 48 genes related to flagella biosynthesis and motility in strain EDL933, homologs of 40 and 35 genes were identified in 13 and seven *E. albertii* strains, respectively (Supplementary Table 1). In strain EDL933, these 48 flagellar genes are distributed at four genomic locations, with a size of 11.5 Kb, 15.6 Kb, 6.7 Kb, and 11.0 Kb for Regions 1–4, respectively. Examining the genomic locations of the flagellar genes in *E. albertii* revealed a similar genes organization as in strain EDL933 (Figure 1A). Among the four genomic locations, the greatest sequence variation was detected in Region 2. In strain EDL933, Region 2 contained seven flagellar genes and eight chemotaxis genes (*cheZYBR*, *tap*, *tar*, and *cheWA*). Unlike strain EDL933, the eight chemotaxis genes were deleted in all *E. albertii* strains examined. Furthermore, an additional deletion of genes *flhBCD* and *motBA* was detected in a subset of *E. albertii* strains including five avian and two clinical strains. This deletion appeared to be mediated by a recombination between the sites within genes *flhA* and *otsA* since a 183-bp *otsA* gene fragment was located immediately upstream of a truncated *flhA* gene. In contrast, the flagellar genes located in the other three regions in strain EDL933 were all conserved in *E. albertii* strains.

Interestingly, a Flag-2 locus, which encodes a secondary flagellar system that resembles the lateral flagella in *Aeromonas hydrophila* and *Vibrio parahaemolyticus* (Ren et al., 2005), was identified in one avian (RM9973) and four clinical *E. albertii* strains. The Flag-2 loci in *E. albertii* varied in size from 33 Kb in the clinical strain 2010C-3449 to 44 Kb in the clinical strain 05-3106. Like the Flag-2 locus in the enteroaggregative *E. coli* (EAEC) strain 042, Flag-2 genes were organized into three gene clusters, separated by the two variable regions, VR1 and VR2 (Figure 1B). In strain 042, the first gene cluster contains 14 genes that are involved in regulation and expression of flagellar basal body components. Homologs of these 14 genes were detected in the avian strain RM9973 and in the clinical strain 05-3106. Genes *lfhB* and *lfiR* were deleted in the strain 07-3866, while genes *lafK* and *lfiEFGHIJ* were deleted in both strains 54-2045 and 2010C-3449 (Supplementary Table 2). The second gene cluster in strain 042 also contains 14 genes encoding flagellar structural proteins and the third gene cluster carries nine genes that are mainly involved in flagellar filament synthesis. Homologs of all genes within the second and the third gene clusters were detected in the Flag-2 positive *E. albertii* strains. In the Flag-2 negative *E. albertii* strains, this region was about 2.5 Kb, containing the truncated two border genes, *flhA* and *lafU* (Figure 1B). A highly similar truncated Flag-2 locus was detected in *E. coli* strains EDL933 and K-12 strain MG1655 (% Identity > 90).

### 3.2 Motility in *E. albertii*

When grown in LB at 30°C for three days, motility was observed in two avian strains, RM10507 and RM10705, and one clinical strain 07-3866 (Table 1). Both strains RM10507 and RM10705 were isolated from brown-headed cowbird and were Flag-2 negative. These three strains remained motile when grown in 5% TSB or in pond water supplemented with 10% pigeon droppings (Table 1). Interestingly, four nonmotile strains when grown in LB, became motile when grown in 5% TSB or in the pond water supplemented with 10% pigeon droppings (Table 1). These four strains included two avian strains, RM9973 and RM9976 that were both isolated from American crow, the chicken isolate 2014C-4356, and the clinical strain 05-3106. The majority of nonmotile phenotypes could be explained by the mutations identified in the flagellar genes, including the deletion of *motAB* and *flhBCD* in avian strains RM15112-RM15116 and in clinical strains 2014C-4015 and 2014EL-1348 (Figure 1B), point deletions in *fliF* of the strains RM9974 and 2011C-4180 and in *motA* of the strain 2013C-4143, and an amber mutation in *flgG* and *flhA* of the strains 54-2045 and 2010C-3449, respectively (Supplementary Table 1).

### 3.3 *E. albertii* fimbrial genes

Homologs of genes encoding 12 fimbriae and pili implicated in adherence, biofilm formation, and pathogenesis in diverse *E. coli* pathotypes were examined in *E. albertii*. All genes are listed in Supplementary Table 3. Homologs of genes encoding curli fimbriae, type 1 fimbriae, and hemorrhagic *E. coli* pilus (HCP) were detected in all strains while homologs of genes encoding adhesive fimbriae, Sfm fimbriae, and P fimbriae were detected in a subset of *E. albertii* strains examined.

#### 3.3.1 Curli genes and expression of curli fimbriae

**FIGURE 2:**
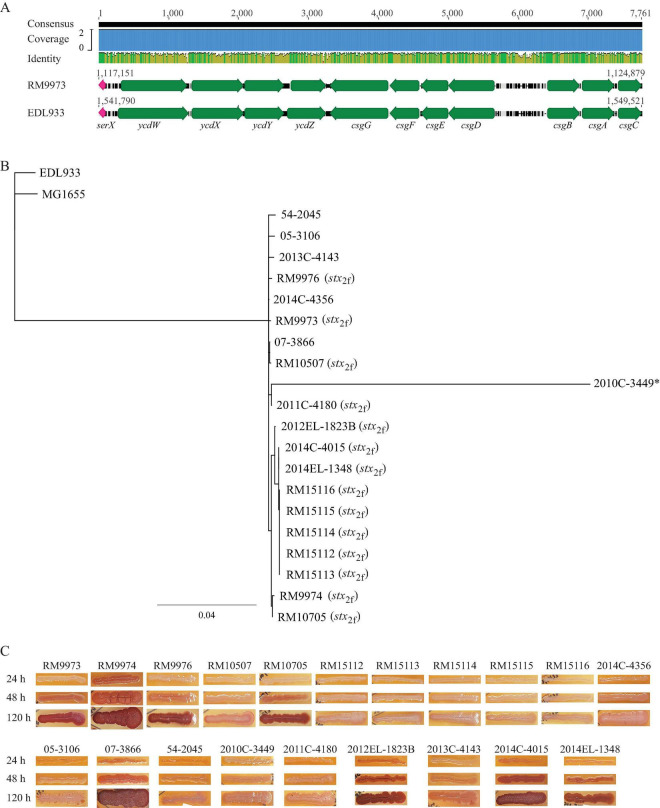
Sequence analyses of curli genes and detection of curli fimbriae in *E. albertii*. **(A)** Chromosomal locations of curli operons and pairwise comparison of the curli genes between the *E. albertii* avian strain RM9973 and *E. coli* O157:H7 strain EDL933. Numbers indicate the corresponding chromosomal positions in each strain. Green arrows represent the annotated genes, and the pink arrows represents tRNA gene *serX*. **(B)** Sequence analysis of curli genes. The curli operons were identified by BLASTn search of a database containing all genomes examined using a 4.4-Kb DNA fragment containing the seven curli genes of the *E. coli* strain MG1655 as a query in Geneious Prime^®^. The sequences of the curli genes were extracted from corresponding genomes and aligned using Clustal Omega alignment in Geneious Prime^®^. A consensus tree was constructed with the following parameters: Genetic Distance Model, Jukes-Cantor; Resampling tree method: Bootstrap; Number of Replicates: 10,000; Support Threshold: 50%. The *stx*_2f_ positive strains are indicated in parentheses. The strain marked with an “*” indicates presence of mutations within the coding sequences of curli genes. **(C)** Detection of curli fimbriae on CRI plates. Curli fimbriae were examined by growing each strain on the CRI plates at 26°C for 24 h, 48 h, and 120 h. Production of curli fimbriae is indicated by red colonies which resulted from the binding of CR dye supplemented in growth medium.

Like *E. coli*, genes related to biogenesis of curli fimbriae in *E. albertii* are organized in two divergent operons, *csgDEFG* and *csgBAC*, and located upstream of tRNA gene *serX* (Figure 2A). Sequence analysis revealed that all *E. albertii* curli genes were placed in the same clade that was separated from the *E. coli* curli genes (Figure 2B). The curli genes of the *E. albertii* strains shared a high sequence similarity ( > 95%) with each other, except for the clinical strain 2010C-3449, in which, both *csgE* and *csgD* were truncated due to an IS insertion, while *csgA* carried an amber mutation.

Unexpectedly, production of curli fimbriae varied greatly among the *E. albertii* strains (Figure 2C). Among the 10 avian strains, production of curli fimbriae was observed in strains RM9973, RM9974, RM9976 and RM10705, although all avian strains carried intact coding sequences for all curli genes. As expected, no curli fimbriae were observed for clinical strain 2010C-3449. Among the other clinical strains, production of curli fimbriae was detected in three out of four *stx*_2f_ positive strains (2012EL-1823B, 2014C-4015, 2014EL-1348) and in the *stx*_2f_ negative strain 07-3866. Colonies of strain 2013C-4143 exhibited pink and light red color following 48 h and 120 h incubation, respectively, suggesting that this strain could produce curli fimbriae under the condition examined but with less amount compared with the other curli-positive strains (Figure 2C).

#### 3.3.2 Type 1 fimbriae genes and expression of type 1 fimbriae

**FIGURE 3:**
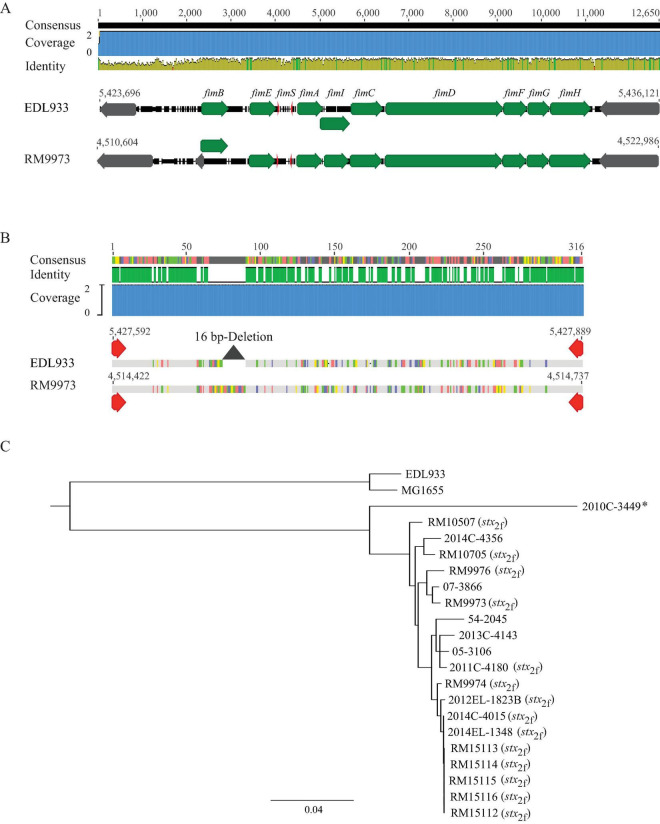
Sequence analyses of *E. albertii* type 1 fimbriae genes. **(A)** Chromosomal locations and pairwise comparison of the type 1 fimbriae genes between the *E. albertii* avian strain RM9973 and *E. coli* O157:H7 strain EDL933. Numbers indicate the chromosomal positions in each strain. Green arrows represent the *fim* genes; Gray arrows represent the neighbor genes; and the red arrows represents right and left inverted repeats (IRs) within *fimS*. The 9-bp IR in strain EDL933 is 5′-ttggggcca-3′ while in strain RM9973, the 9-bp IR is 5′-ttgggacca-3′. **(B)** Pairwise alignment of the *cis* element *fimS* of the *E. albertii* avian strain RM9973 and *E. coli* O157:H7 strain EDL933. Red arrows represent the IRs. The grey triangle represents the 16 bp-deletion in EDL933 *fimS*. **(C)** Sequence analysis of *E. albertii* type 1 fimbriae genes with the *fim* genes in *E. coli* strains EDL933 and MG1655. The *fim* genes were identified by BLASTn search of a database containing all genomes examined in this study using an 8.7-Kb DNA fragment containing the nine *fim* genes of the *E. coli* strain EDL933 as a query in Geneious Prime^®^. The sequences of the *fim* genes were extracted from corresponding genomes and aligned using Clustal Omega alignment in Geneious Prime^®^. A consensus tree was constructed using Geneious Tree Builder with the following parameters: Genetic Distance Model, Jukes-Cantor; Resampling tree method: Bootstrap; Number of Replicates: 10,000; Support Threshold: 50%. The *stx*_2f_ positive strains are indicated in parentheses. The strain marked with an “*” carries mutations within the coding sequences of type 1 fimbriae genes.

In *E. coli*, the type 1 fimbriae genes (*fimB*, *fimE*, and *fimAICDFGH*) are located on an 8.8-Kb DNA fragment. Expression of the type 1 fimbriae is controlled by a phase variation mechanism, in which, transcription of *fimA* is switched to “ON” or “OFF” by an invertible DNA element, *fimS*, and two site-specific recombinases encoded by genes *fimB* and *fimE*, respectively (Figure 3A). Homologs of the nine *fim* genes were identified in all 20 *E. albertii* strains examined. Additionally, the invertible element, *fimS*, flanked by two 9-bp inverted repeats (IRs), was also detected in all *E. albertii* strains. The IRs (5′-TTGGGGCCA-3′) in *E. albertii* strains were identical to the IRs in *E. coli* strains EDL933 and K-12 strain MG1655, except for the avian strain RM9973, in which a single base substitution of G to A occurred at position 6.

Expression of type 1 fimbriae in *E. coli* O157:H7 strains including EDL933 is silenced due to a 16 bp deletion in *fimS* (Roe et al., 2001). This deletion locks the transcription of *fimA* at the “OFF” orientation. Comparative analyses of *E. albertii fimS* genes with the EDL933 *fimS* revealed an intact *fimS* in *E. albertii* strains, like the *fimS* in *E. coli* K-12 strain MG1655 (Figure 3B). Sequence analyses of other *fim* genes placed all *E. albertii* strains in the same clade, separated from the *fim* genes in the *E. coli* strains (Figure 3C). Various mutations including point deletions and IS insertions were revealed in the *fimA, fimC, and fimD genes* of clinical strain 2010C-3449, the *fimD* of the clinical strain 54-2045, and the *fimI* of the clinical strain 2013C-4143 (Supplementary Table 3).

However, a mannose-sensitive hemagglutination assay failed to detect type 1 fimbriae in any of the 20 *E. albertii* strains examined when they were grown in LBHS at 37°C or in LBNS at 28°C, like strain EDL933. Production of type 1 fimbriae was detected in *E. coli* DH5a cells under both testing conditions.

#### 3.3.3 Other fimbriae genes

Homologs of genes encoding hemorrhagic *E. coli* pilus (*hcpABC*) were identified in all 20 *E. albertii* strains, and mutations in *hcpB* (annotated as *gspE* in *E. albertii*), encoding the type II secretion system protein GspE, were present in seven out of the 20 strains examined (Supplementary Table 3). Similarly, homologs of genes encoding the Sfm fimbriae (*sfmACDHF* and *sfmZ*) were identified in the 18 out of the 20 strains examined. Mutations were most common in *sfmD*, encoding a fimbrial biogenesis usher protein (Supplementary Table 3). In the clinical strains 54-2045 and 2010C-3449, only a homolog of *sfmA* was present. Among the adhesive fimbriae genes examined, homologs of *cfaABCD* genes, which are often present on the chromosomes of EHEC strains, were identified in 10 out of the 14 stx_2f_-positive *E. albertii* strains, while homologs of *faeCDEFGHIJ* genes, which are often present on the plasmids of enterotoxigenic *E. coli* (ETEC) strains, were identified in the clinical strain 07-3866 and in the chicken isolate 2014C-4356 (Supplementary Table 3). In *E. coli*, there are 12 genes (*papXGFEKJDCHABI*) related to biogenesis of P fimbriae. Homologs of seven genes, *papEKJDCHA*, were identified in seven out of the 20 *E. albertii* strains, homologs of five genes, *papJDCHA*, were identified in the chicken isolate 2014C-4356, and homologs of four genes, *papDCHA*, were identified in the clinical strain 2012EL-1823B.

### 3.4 Nonfimbrial adhesin genes and their genetic diversity

The most common autotransporter adhesin genes detected in *E. albertii* were *paa*, *ehaC*, *eaeH*, *ehaB*, and *sinB* (Supplementary Table 3). *paa* encodes an AcfC family adhesin. A homolog of *paa* was present in nearly all *E. albertii* strains examined and exhibited > 80% sequence identity with the *paa* gene in EHEC strain EDL933. The gene *ehaC* encodes an AIDA-I family autotransporter adhesin. A homolog of *ehaC* was detected in all *E. albertii* strains examined, although a point deletion and a point insertion were present in the avian strain RM10705 and the clinical strain 2010C-3449, respectively (Supplementary Table 3). The *E. albertii ehaC* genes exhibited ∼ 75% sequence identity with the *ehaC* gene in strain EDL933. *eaeH* encodes an intimin-like adhesin FdeC. A distant homolog (∼ 80% length in CDS and 27.6% identity in amino acids) was identified in all *E. albertii* strains examined, although mutations were detected in the clinical strain 2011C-4180 (IS insertion), and avian strains RM10507 (Insertion of 5’-GTCTG-3’) and RM10705 (a point deletion). A homolog of *ehaB*, ranging in size from 2430 bp to 2979 bp was detected in *E. albertii* strains. Interestingly, the *ehaB* genes in avian strains RM9973 and RM9976, and in clinical strains 05-3106, 54-2045, and 2014C-4356, displayed higher sequence similarity with the EDL933 *ehaB* gene compared with the *ehaB* genes in other *E. albertii* strains. The gene *sinH*, encoding an intimin-like inverse autotransporter, was present in all *E. albertii* strains examined. In fact, the gene *sinH* appeared to be widespread in *E. albertii* but only present in a subset of *E. coli* strains. Homologs of *sinH* were not identified in EHEC strain EDL933.

### 3.5 Biofilm formation

**FIGURE 4:**
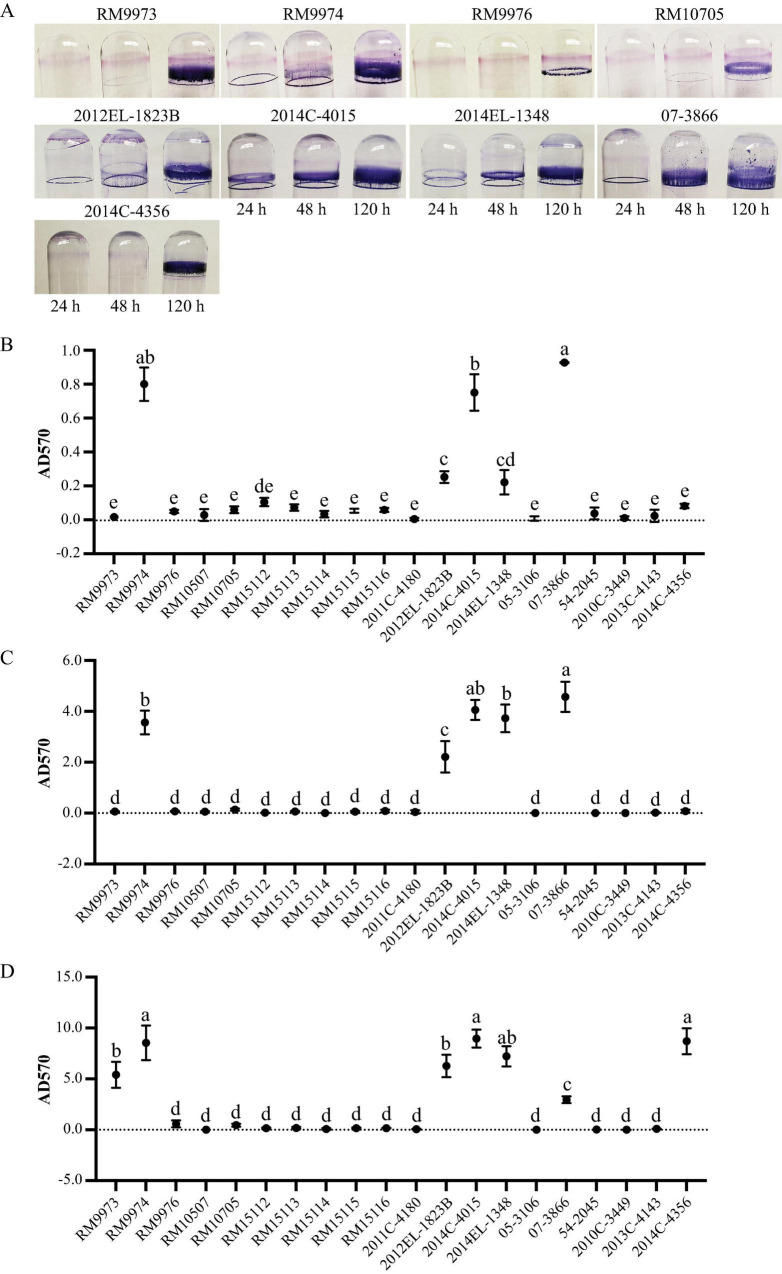
Biofilm formation by *E. albertii* strains on glass surfaces. **(A)** Crystal violet staining the attached biomass on the glass surfaces under a static growth condition for 24 h, 48 h, and 120 h. Only strains that can produce visible rings are shown here. **(B–D)** quantitative analyses of biofilms under a static growth condition for 24 h **(B)**, 48 h **(C)**, and 120 h **(D)**. Each data set represents the mean and SD of three biological replicates. Differences that are statistically significant (One-way ANOVA followed by a Tukey’s multiple comparisons test, adjust *P* < 0.05) are indicated by different letters. The detailed results of the statistical analyses are presented in Supplementary Table 4.

Following the initial 24 h incubation, a visible ring was observed for avian strain RM9974 and four clinical strains, 2012EL-1823B, 2014C-4015, 2014EL-1348, and 07-3866 (Figure 4A). Consistently, quantitative analysis revealed that the attached biomass for the above five strains were all significantly greater than the rest of the strains except the comparison between strains 2014EL-1348 and RM15112 (One-way ANOVA, adjust *P* < 0.05) (Supplementary Table 4). Among the five biofilm producing strains, strains RM9974, 2014C-4015, and 07-3866 produced significantly greater amounts of biofilm than the other two strains (Figure 4B). Following 48 h incubation, the attached biomass for the five biofilm producing strains were all significantly greater than the corresponding biofilms at 24 h (Figure 4C) (One way ANOVA test, adjust *P* < 0.05). Among the strains that did not produce any detectable biofilm at 24 h, a visible ring was observed for strains RM9973 and RM10705 (Figure 4A). Quantitative analysis revealed that strains 07-3866, 2014C-4015, 2014EL-1348, and RM9974 produced significantly greater amounts of biofilm than that of the strain 2012EL-1823B (One way ANOVA test, adjust *P* < 0.05) (Figure 4C). Among the strains that did not produce any visible biofilms at 48 h, strain 2014C-4356 produced a considerable amount of biomass on the glass surface following 120 h incubation (Figure 4A). Among the strains that produced biofilms following 48 h incubation, a significant increase in attached biomass was observed for all strains following 120 h incubation (One-way ANOVA, adjust *P* < 0.05) (Figure 4D). At 120 h post inoculation, quantitative analysis revealed that avian strain RM9974, chicken isolate 2014C-4356 along with the clinical strain 2014C-4015 were the strongest biofilm producers, followed by the clinical strains 2014EL-1348 and 2012EL-1823B, and the avian strain RM9973, which all produced significantly greater amounts of biofilm than the clinical strain 07-3866 (One-Way ANOVA, adjust *P* < 0.05). For avian strains RM9976 and RM10705, although a visible ring was detected on glass surfaces, they were not significantly different from those of non-biofilm producing strains (Figure 4D).

## 4 Discussion

Flagellar motility allows bacteria to move rapidly towards nutrients and away from toxic substances, thus it plays an essential role in bacteria to explore new niches and to establish colonization. Moreover, flagella also serve as a virulence factor in many enteric pathogens, contributing to adhesion, invasion, and host colonization (Moens and Vanderleyden, 1996; Colin et al., 2021). In *E. coli*, nearly 50 genes are involved in flagella assembly and function. Expression of the flagellar genes is tightly regulated by a three-tiered transcriptional hierarchy to ensure production of flagellum at the right time and under the applicable conditions (Khan et al., 2020). The group I genes, *flhDC*, encode the master transcriptional regulator FlhDC that activates the expression of the group II genes. In *E. coli*, there are nearly 30 genes belonging to the group II and many of these genes encode components of the flagellum basal body and hook and the sigma factor 28 FliA. FliA regulates the expression of group III genes, which are involved in synthesis of complete flagellum and chemotaxis systems. The regulation of flagellar gene expression in *E. albertii* is unknown, although a similar hierarchical regulation fashion is expected considering the close phylogenetic relationship between the two species. Originally, *E. albertii* was thought nonmotile and lacked flagella although 74% *E. albertii* strains were reported to carry a complete set of flagellar biosynthesis genes (Abbott et al., 2003; Ooka et al., 2015). Induction of flagellar motility by low osmotic pressure was observed in 27 out of the 59 *E. albertii* strains tested (Ikeda et al., 2020); similarly, induction of swimming motility by nutrients derived from pigeon droppings was observed in six out of the 12 strains examined (Murakami et al., 2020), implying strain variation in expression of flagellar motility in *E. albertii*. Consistently, our study revealed great diversity in flagellar genes repertoire and conditionally expressing swimming motility in *E. albertii*. Among the 20 *E. albertii* strains examined, three were motile regardless of the growth conditions, while four were motile only when grown in pond water supplemented with pigeon droppings or in the diluted TSB medium. Deletion of a large DNA fragment containing genes *flhAB*, *motBA*, and *flhCD* is likely the molecular basis of nonmotile phenotypes observed in five avian and two clinical strains. Furthermore, a loss-of-function mutation in genes *fliC, fliF*, *fliJ*, *flgD*, *flgG*, *flhA*, and *motA* may explain some but not all non-motile phenotypes observed in our study. Considering the highly complex regulation in flagellar gene expression, comparative transcriptomic studies may provide insight into the molecular basis of the strain variation in expression of flagellar motility.

Our study revealed the presence of the Flag-2 locus in *E. albertii*. The Flag-2 locus appears to be widespread among the *Enterobacterales* (De Maayer et al., 2020) and serve as a hot spot for gene insertions and deletions. Consistently, great sequence variation was observed among the five Flag-2 loci identified, including large deletions, point mutations and transposon insertions. However, several VR1 genes that are predicated on having a role in posttranslational regulation of flagellar biosynthesis are conserved in *E. albertii*, including the glycosyltransferase gene and the lysine-N-methylase gene. Unlike the primary flagellar system, the function of the Flag-2 locus is not fully understood. Expression of Flag-2 genes was observed in *Yersinia enterocolitica* with a maximal level at 20°C and, in *Plesiomonas shigelloides*, the Flag-2 locus encoded lateral flagella appeared to be essential for swarming motility (Bresolin et al., 2008; Merino et al., 2015). Systematic analyses of cargo genes located in the VR2 in Flag-2 loci suggested a role in secretion of virulence factor and in inter-bacterial competition (De Maayer et al., 2020). Searching other *E. albertii* genomes deposited in public databases as of April 2024 revealed that about 25% of genomes carry a Flag-2 locus. Additional studies are needed to elucidate any physiological roles or ecological benefits conferred by this secondary flagellar system in *E. albertii*.

Among the 12 fimbriae/pili that are commonly present in *E. coli*, genes encoding curli fimbriae, hemorrhagic *E. coli* pilus, type 1 fimbriae, and Sfm fimbriae were identified in most of the *E. albertii* strains examined, while genes related to biogenesis of adhesive fimbriae, or P fimbriae were only present in a subset of strains. Curli, also known as bacterial amyloid, is an important colonization factor involved in initial surface attachment, biofilm formation, and induction of the host inflammatory response (Barnhart and Chapman, 2006). Although 19 out of the 20 *E. albertii* strains examined in our study carried intact curli genes, production of curli fimbriae was detected in only nine strains. This strain variation could not be explained solely by the differences in the coding sequences of the curli genes or the differences in the intergenic regions between the two curli operons, including the promoters of *csgD* and *csgB*, since some curli-deficient strains shared the identical intergenic sequences with the curli expressing strains (Data not shown). Strain variation in curli production were reported in *E. coli* and *Salmonella enterica* (Romling et al., 1998; Dyer et al., 2007), which both have served as the model organisms for studying curli biogenesis and regulation. In both *E. coli* and *S. enterica*, expression of curli is regulated by a complex regulation network involving multiple transcriptional regulators, two-component regulatory systems, and in some isolates cyclic dinucleotide 3’,5’-cyclic di-GMP (Barnhart and Chapman, 2006; Blomfield and van der Woude, 2007). Therefore, mutations in any of these regulators or the target sequences that interact with the regulators directly or indirectly could have an impact on the expression of curli fimbriae. For example, mutations in genes encoding the transcriptional regulators *rpoS* or *rcsB* were reported to be the molecular bases of strain variation in curli production in EHEC O157:H7 (Carter et al., 2012; Carter et al., 2014) and mutations in the *csgD* promoter could lead to overproduction of curli fimbriae (Uhlich et al., 2002). Knowledge about the regulation of curli fimbriae in *E. albertii* is limited. Examining genes encoding putative transcriptional regulators of the curli genes in *E. albertii* revealed a loss-of-function mutation in the *rpoS gene* in strains 05-3106 and 54-2045, while no mutations were identified in genes encoding Crl, MlrA, CpxRA, OmpR-EnvZ, or RcsBC. Additional studies are required to gain a comprehensive understanding of curli regulation network in *E. albertii* as well as the environmental and physiological signals that may induce or repress the expression of curli fimbriae.

Although the majority of *E. albertii* strains examined in our study carried functional *fim* genes and an intact *fimS*, none of them displayed a mannose-sensitive hemagglutination (MSHA) phenotype under the growth conditions examined. Expression of type 1 fimbriae was reported to be dependent on the growth conditions. For example, optimal production of a predominantly type 1 fimbriae positive population in *Shigella* required serial passage every 48 to 72 h in unshaken brain heart infusion broth at 37°C (Snellings et al., 1997). Since the goal of our study was to reveal if the type 1 fimbriae contributed to the biofilm formation in *E. albertii*, the conditions tested for production of type 1 fimbriae were the conditions used for examining biofilm formation, which may not be optimal for expression of type 1 fimbriae. Additionally, the phase variation of type 1 fimbriation is regulated at multiple levels. In *E. coli*, switch of *fimS* is required but not sufficient for biosynthesis of type 1 fimbriae. Besides FimB and FimE, other transcriptional regulators including IHF, Lrp, and H-NS were reported to be involved in *fimS* switch (Blomfield and van der Woude, 2007). Variations in the activities of FimB and FimE, cross talks between fimbrial operons, as well as the presence of other recombinases can all contribute to variation in expression of type 1 fimbriae. Additional studies are required to understand growth conditions, physiological cues, and environmental signals for induction of type 1 fimbriae in *E. albertii*.

Among the nonfimbrial adhesins examined, genes encoding the autotransporter (AT) adhesins were predominant in *E. albertii*. For example, homologs of *ehaA*, *ehaB*, *ehaC*, and *upaH* that all encode an AIDA-I type autotransporter (AT) adhesin were identified in all or most of the strains examined and a homolog of *ehaG*, encoding a trimeric AT adhesin was identified in all strains. Other AIDA-1 type adhesins genes in *E. albertii* included *aatA*, *aidA*, *agn43*, and *cah*. In *E. coli*, Ag43 is the most prevalent AIDA-1 type AT adhesin, however, in *E. albertii*, the *agn43* was identified only in a clinical strain. Other commonly detected non-fimbrial adhesin genes were *eaeH*, *paa*, and *sinH*. The gene *eaeH* encodes an intimin-like adhesin that facilitates adhesion of bacterial cells, delivery of heat-labile toxin, and colonization of the small intestine in ETEC (Sheikh et al., 2014). The gene *paa*, encoding an AcfC family adhesin, is widespread in both EHEC and ETEC strains. Paa contributes to the formation of A/E lesions in animal hosts and thus is an important virulence factor in various *E. coli* pathotypes (An et al., 1999; Batisson et al., 2003). The *paa* gene appears to be widespread in *E. albertii* and in some strains, there are two *paa* loci, including the bird strains isolated in Poland (BioSample numbers: SAMN33094111, SAMN33094114, SAMN33094099, SAMN33094102, and SAMN33094112), and a poultry strain isolated in China (SAMN17525956). The gene *sinH* encodes an intimin-like inverse autotransporter. The inverse autotransporters were reported to play a role in biofilm formation in *E. coli* and contributed to biofilm formation and virulence in *Yersinia ruckeri* (Martinez-Gil et al., 2017; Goh et al., 2019; Wrobel et al., 2020). However, deletion of *sinH* in the UPEC strain CFT073 did not impact the biofilm formation significantly, rather, the mutant displayed a significant fitness reduction during UTI in a murine model (Shea et al., 2022). Like *paa*, *sinH* appears to be conserved in *E. albertii*. BLASTn search of additional 57 complete *E. albertii* genomes deposited in GenBank as of April 2024 identified a *sinH* in all of them.

Like curli production, *E. albertii* strains differed greatly in biofilm formation on glass surfaces. Consistent with our previous report that, in STEC, curli fimbriae are important for biofilm formation on abiotic surfaces (Carter et al., 2016; Carter et al., 2019), all curli-producing *E. albertii* strains produced moderate or strong biofilms under the condition examined. Interestingly, some curli-positive strains produced visible biofilm following a 24-h incubation, while others did not produce biofilms until a 120-h incubation, implying a difference in biofilm development among the *E. albertii* strains. Furthermore, although no curli fimbriae were detected in chicken isolate 2014C-4356, strong biofilms were observed on day 5 of incubation, suggesting a role of other adhesins in biofilm formation. Strains 2014C-4356 and 07-3866 were the only strains carrying genes encoding the adhesive fimbriae that are located on a large plasmid commonly found in ETEC strains, such as pUMNK88_Hly (GenBank accession # NC_017643.1). It requires further investigation to determine if the plasmid-borne adhesive fimbriae are expressed in *E. albertii* and whether it contributes to biofilm formation in strains that do not produce curli fimbriae.

Biofilms of foodborne pathogens can enhance their survival and persistence in diverse ecological niches and serve as sources of contamination in food production environments and of infection in health-care environments. Adhesion is the first step in biofilm development and in establishing colonization in animal hosts. Strong adherence often implies enhanced surface attachment and biofilm formation, leading to increased fitness and pathogenic potential. Therefore, understanding the adhesion capability and the underlining factors in *E. albertii* would provide valuable information for development of effective control strategies. Our study revealed that curli fimbriae, Type 1 fimbriae, Sfm fimbriae, and HCP appear to be the common fimbrial adhesins in *E. albertii*, while adhesive fimbriae was a strain-specific trait. Among the numerous nonfimbrial adhesins identified in *E. albertii*, autotransporter adhesins EhaA, EhaB, EhaC, EhaG, and SinH, and the adherence factors EaeH and Paa are common, while Agn43, Cah, and Iha that are widespread in *E. coli*, are only associated with a few strains. *E. albertii* strains carry different combinations of fimbrial and nonfimbrial adhesins that may facilitate colonization of *E. albertii* in diverse niches. Our study further revealed great variations in expression of curli fimbriae and in biofilm production, suggesting complex regulation in expression of adhesins in *E. albertii*. Studies are needed to identify environmental cues that induce the adhesions expression and the receptors specifically interacted with each adhesin to gain insight into molecular basis of niche selection for *E. albertii*, an emerging human and avian pathogen.

## Data Availability

The datasets presented in this study can be found in online repositories. The names of the repository/repositories and accession number(s) can be found in the article/Table 1.

## Supplementary material

Supplementary Table 1 *E. albertii* primary flagellar genes.

Supplementary Table 2 *E. albertii* secondary flagellar genes.

Supplementary Table 3 *E. albertii* fimbrial and non-fimbrial adhesin genes.

Supplementary Table 4 Statistical analyses of *E. albertii* biofilms.
